# Targeting the p53–MDM2 interaction to treat cancer

**DOI:** 10.1038/sj.bjc.6602164

**Published:** 2004-09-28

**Authors:** C Klein, L T Vassilev

**Affiliations:** 1 Pharma Research, Roche Diagnostics GmbH, Penzberg D-82372, Germany; 2 Discovery Oncology, Hoffmann-La Roche Inc., Nutley, NJ 07110, USA

**Keywords:** p53, MDM2, protein–protein interaction, small molecule

## Abstract

The tumour suppressor p53 is a transcription factor with powerful antitumour activity that is controlled by its negative regulator MDM2 (mouse double minute 2, also termed HDM2 in humans) through a feedback mechanism. MDM2, which is overproduced in many tumours, binds p53 and inhibits its function by modulating its transcriptional activity and stability. Activation of p53 in tumour cells by inhibiting its physical interaction with MDM2 has been in the focus of cancer drug discovery. However, development of nonpeptidic MDM2 antagonists turned out to be challenging. Recently, the first potent and selective small-molecule antagonists of MDM2, the Nutlins, have been identified. Studies with Nutlins provided *in vitro* and *in vivo* proof-of-principle for targeting p53–MDM2 interaction for cancer therapy.

## 

Since the discovery of its powerful growth suppressive and proapoptotic activity, the tumour suppressor p53 has been in the centre of attention of drug hunters. The idea of unleashing the destructive powers of p53 inside cancer cells has become even more attractive after the realisation that p53 is controlled largely by a single master regulator, MDM2, which binds the tumour suppressor and negatively modulates its activity and stability. Therefore, MDM2 antagonists able to release p53 from the inhibitory grip of MDM2 are expected to stabilise and activate the tumour suppressor, leading to cell cycle arrest or programmed cell death (apoptosis) of cancer cells. Such antagonists could represent a novel modality to treat tumours in which p53 has retained its wild-type structure and function.

Targeting the physical interaction between p53 and MDM2 has been regarded as the most direct of all p53-activating strategies. However, protein–protein interactions have long been viewed as high-risk targets due to the fact that proteins generally offer relatively large and flat interacting surfaces that are not readily disturbed by small-molecule drugs (Arkin and Wells, 2004). This view has been challenged during the recent years by several proof-of-concept studies that targeted protein interactions with peptidomimetic and small-molecule antagonists (Toogood, 2002; Arkin and Wells, 2004). Most of these compounds, however, have exhibited low potency and/or inadequate pharmacological properties and the development of small-molecule protein–protein therapeutics is still in its infancy.

The strong interest in targeting the p53–MDM2 interaction has led to the identification of several macromolecular tools that helped to validate the principle of this p53-activating approach. However, the discovery of pharmacologically relevant small-molecule inhibitors of p53–MDM2 binding turned out to be more challenging than initially thought. Nevertheless, progress in this direction has been made and recently the first potent and selective low-molecular-weight nonpeptidic MDM2 antagonists have been identified. This minireview focuses on the p53-MDM2 interaction as a target for discovery of novel cancer therapeutics.

### Cellular roles of p53 and mdm2

The tumour suppressor p53 plays a pivotal role in protection from cancer development that may arise from diverse forms of cellular stress (Levine, 1997; Vogelstein *et al*, 2000; Vousden and Lu, 2002). p53 is a potent transcription factor, which is activated following stress and regulates multiple downstream genes implicated in cell cycle control, apoptosis, antiangiogenesis and senescence. Owing to its central role as a cellular gatekeeper, the p53 pathway is the most frequent target of genetic alterations in cancer. Approximately half of all human tumours express p53 that is disabled by mutations in its DNA-binding domain and is thus inactive as a transcription factor (Hainaut and Hollstein, 2000). In addition, p53 can be inactivated by binding to viral proteins such as the E6 protein of papilloma viruses (Levine, 1997). Growth suppressive and proapoptotic activity of p53 could harm proliferating cells that are not under stress. Therefore, the level of p53 in these cells is a subject of tight control. This important role is played by the p53 regulator MDM2 (mouse double minute 2, also termed HDM2 in humans).

MDM2 was originally identified as an oncoprotein that binds to p53 and inhibits p53-mediated transactivation (Momand *et al*, 2000). The *mdm2* gene was found to be upregulated in human tumours and tumour cell lines by gene amplification, increased transcript levels and enhanced translation. The overall frequency of MDM2 amplification in human tumour tissue samples is approximately 7% with the highest frequency observed in soft-tissue sarcomas (20–30%), osteosarcomas (16%) and oesophageal carcinomas (13%). Simultaneous mutation of p53 and amplification of MDM2 does not generally occur within the same tumour, suggesting that MDM2 amplification is an effective means for inactivation of p53 function (Momand *et al*, 1998).

*The mdm2* gene encodes a protein consisting of several domains: (i) N-terminal domain that contains the binding sites for p53, p73 and E2F; (ii) acidic domain interacting with the tumour suppressor p14^ARF^; (iii) putative Zn-finger and binding site for the retinoblastoma protein Rb; and (iv) a RING-finger and E3 ligase domain that is responsible for the ubiquitination of p53. In addition, MDM2 contains nuclear import and export sequences (Momand *et al*, 2000).

The main cellular function of MDM2 is to regulate p53 levels. This is strongly supported by the observation that embryonic lethality of *mdm2*-null mice can be reversed only by the simultaneous deletion of the p53 gene (Jones *et al*, 1995; Monies de Oca Luna *et al*, 1995). MDM2 regulates p53 through an autoregulatory feedback loop. When nuclear p53 level is elevated, it activates the transcription of the *mdm2* gene, thus raising the level of MDM2 protein. In turn, MDM2 binds to p53, which (a) blocks its N-terminal transactivation domain and (b) targets p53 for degradation via the ubiquitin–proteasome system following ubiquitinylation through its E3 ligase activity. Both p53 and MDM2 have a short half-life and their nuclear concentrations are kept at very low levels as a result of the proper functioning of the regulatory circuit (Freedman *et al*, 1999; Juven-Gershon and Oren, 1999). However, in cancers overexpressing MDM2 this feedback loop is dysregulated. Stress-induced p53 activation mechanisms in these tumours are believed to be inadequate, leading to inefficient growth arrest and/or apoptosis. Therefore, blocking the p53–MDM2 interaction is expected to overcome the oncogenic consequences of MDM2 overproduction and to restore p53 function. Treatment of cancer cells with MDM2 antagonists should result in the concurrent transcriptional activation of p53 downstream genes followed by the induction of cell cycle arrest and apoptosis.

### The p53–MDM2 interaction

Genetic and biochemical studies mapped the p53–MDM2 interaction sites to the N-terminal domain of MDM2 and the N-terminal part of the transactivation domain of p53 also termed BOX1 domain (Chen *et al*, 1993; Picksley *et al*, 1994). Crystal structures of the N-terminal domains of human and *Xenopus laevis* MDM2 in complex with short peptides from the N-terminal domain of p53 (residues 15–29) revealed the structural basis of the interaction between p53 and MDM2 (Kussie *et al*, 1996). Upon binding to MDM2, the unstructured p53 transactivation domain (Lee *et al*, 2000; Dawson *et al*, 2003) forms an amphiphilic a-helix that projects the hydrophobic residues of Phe^19^, Trp^23^ and Leu^26^ into a deep hydrophobic binding pocket on the MDM2 surface (Kussie *et al*, 1996; Blommers *et al*, 1997). The p53 structural changes induced by MDM2 provide an explanation for how MDM2 binding inhibits the transcriptional activity of p53. Mouse double minute 2 binding site on p53 coincides with the *α*-helical FxxΦΦ-motif (F: phenylalanine; Φ: hydrophobic residue; in p53: Trp^23^) that is responsible for binding of important components of the transcription machinery such as hTAFII31 (Uesugi and Verdine, 1999).

Searching for novel peptides that can bind MDM2, Bottger *et al* have identified the IPS peptide displaying a 30-fold higher affinity than the native p53 peptide (17–29) (Bottger *et al*, 1996). This peptide contained several additional hydrophobic residues. Alignment of the peptide sequences derived from phage display allowed the definition of a consensus motif for binding to MDM2 (PxFxDYWxxL) (Bottger *et al*, 1996; Bottger *et al*, 1997a). Combinatorial permutation and biophysical studies using synthetic peptides have revealed the main structural requirements for binding to MDM2 (Schon *et al*, 2004). These experimental data together with rationalisation on the basis of the crystal structure of p53–MDM2 interaction (Kussie *et al*, 1996) have allowed to develop a quantitative structure–activity relationship (QSAR) that might help in the identification of small-molecule MDM2 antagonists (Galatin and Abraham, 2001).

Using a semirational drug design and NMR spectroscopy, Garcia-Echeverria *et al* (2000) generated a highly potent peptidic MDM2 antagonist termed AP peptide (19–26). The affinity of the AP peptide has been enhanced by the introduction of artificial amino acids in the minimal sequence derived from the IP3 peptide. These residues have stabilised entopically the helical conformation of the peptide and formed additional polar and hydrophobic van der Waals interactions with MDM2. This optimisation improved the affinity of the AP peptide to MDM2 by 60-fold in comparison with IP3 and almost 2000-fold in comparison with the native p53 peptide (Garcia-Echeverria *et al*, 2000). Recently, cyclic peptidomimetic inhibitors of MDM2 have been described that mimic the *α*-helix by *β*-hairpins (Fasan *et al*, 2004).

### Validation of the p53–MDM2 interaction as a drug target

The realisation that p53–MDM2 binding involves the interaction of three critical amino-acid residues from p53 with a well-defined hydrophobic pocket on the surface of MDM2 have raised the hope that identifying pharmacological inhibitors of this interaction might be possible. Several different approaches have been used to validate p53–MDM2 interaction as a drug target. These include: (i) microinjection of monoclonal antibodies directed against the p53 binding site on MDM2 (Blaydes *et al*, 1997; Bottger *et al*, 1997b); (ii) inhibition of MDM2 expression by antisense oligonucleotides (Chen *et al*, 1999; Wang *et al*, 1999, 2001; Geiger *et al*, 2000; Zhang *et al*, 2003); (iii) microinjection or intracellular expression of short fusion proteins (Bottger *et al*, 1997b; Wasylyk *et al*, 1999); and (iv) transduction of cells with IP3 and AP peptides coupled to peptide transduction domains (e.g. penetratin and Tat) (Chene *et al*, 2000, 2002; Garcia-Echeverria *et al*, 2001). Collectively, these studies have demonstrated that blocking the p53–MDM2 binding can disrupt the p53 regulatory circuit, leading to p53 accumulation and activation of the p53 pathway. However, most of the studies have used macromolecular tools (proteins or peptides) with limited applicability to studies in living cells or animal models of cancer. One notable exception is the antisense approach. Several antisense studies have demonstrated that phosphorothioate oligonucleotides directed against MDM2 can suppress MDM2 expression effectively, thus leading to the accumulation of p53 and activation of the p53 pathway. The consequences of p53 activation have been examined in several mouse xenograft models of human cancer. They have shown the anticipated tumour growth suppression that is enhanced by combination with established genotoxic drugs (Zhang *et al*, 2003). Surprisingly, in all antisense studies the antitumour effect was not limited to cancer cells with wild-type p53 as expected from disruption of the p53–MDM2 regulatory loop. Instead, antisense treatment affected equally well cells in which p53 is disabled by mutation. These observations have complicated the interpretation of the antisense results. However, a recent finding from the same group that MDM2 can bind the cyclin-dependent kinase inhibitor p21^waf1/cip1^ and inhibit its activity have shed a new light on the role of MDM2 and the consequences of MDM2 inhibition (Zhang *et al*, 2004).

An important aspect in the validation of the p53–MDM2 interaction as a cancer target is assessing the consequences of p53 activation in the normal proliferating tissues. It has been shown that activation of the p53 pathway can lead to toxicity in tissues with relatively high proliferative index. This toxicity could arise from both main functions of p53: cell cycle arrest and apoptosis, and could narrow the therapeutic window of any p53-activating therapy. Indeed, MDM2-null mouse embryos die at day 5.5 most likely due to apoptosis in the rapidly proliferating tissues (de Rozieres *et al*, 2000). However, recent studies both *in vitro* and *in vivo* have suggested that the consequences of p53 activation in cancer and normal cells may differ. Activation of the p53 pathway in human fibroblasts has been shown to elicit growth arrest when cancer cells tend to respond with induction of apoptosis (Smart *et al*, 1999). Studies with conditional MDM2 knockouts provide *in vivo* evidence for the rationale of inhibiting the p53–MDM2 interaction for the treatment of cancer (Mendrysa *et al*, 2003). According to these studies, inhibition of MDM2 does not cause major target-related toxicity with the possible exception of certain mild haematopoietic abnormalities. Moreover, they imply that even partial inhibition of MDM2 is sufficient to activate the p53 pathway *in vivo* and/or synergise with radiation or cytotoxic therapeutics. Genetic modulation of MDM2 levels in mice also suggests that p53 regulation during homeostasis may differ from its regulation in cancer tissues (O'Leary *et al*, 2004).

### Pharmacological inhibitors of the p53–MDM2 interaction

In this section, we will review the efforts on developing direct protein–protein binding antagonists of MDM2. For an example of targeting the E3 ubiquitin ligase activity of MDM2 see Lai *et al* (2002). The first reported small-molecule MDM2 antagonists, the chalcones, are derivatives of phenoxy acetic acid and phenoxymethyl tetrazole (Figure 1A

**Figure 1:**
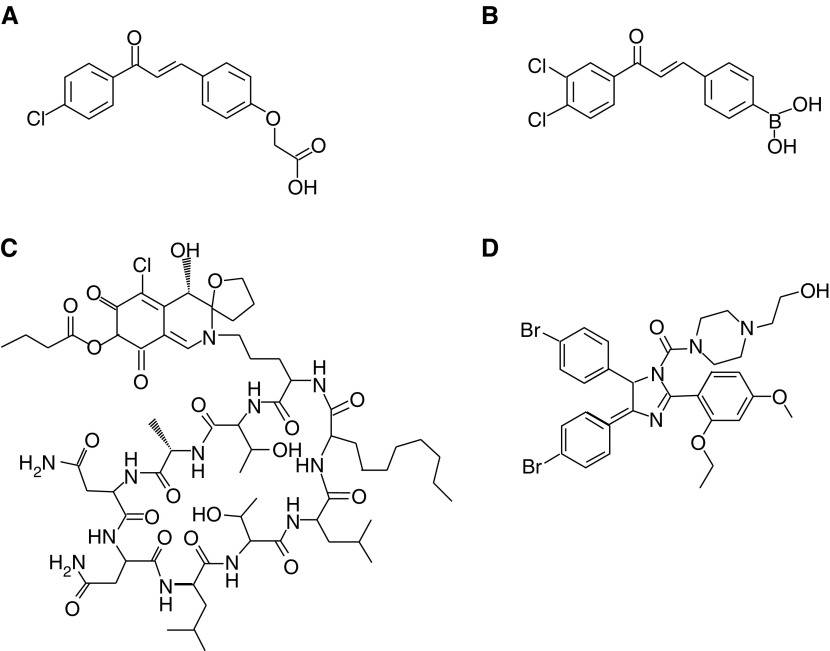
Small-molecule inhibitors of p53–MDM2 binding. (**A**) Chalcone, (**B**) boronic–chalcone, (**C**) Chlorofusin, and (**D**) Nutlin-2.

). Chalcones have been shown to inhibit p53–MDM2 interaction with IC_50_ values in the high *μ*M range by binding to the p53 pocket on MDM2 as revealed by NMR spectroscopy. However, in addition to their low potency they have shown other liabilities such as inhibition of glutathione-*S*-transferase activity that have limited severely their use (di Domenico *et al*, 1999; Stoll *et al*, 2001). Recently, novel boronic–chalcone derivatives have been described as putative MDM2 antagonists with antitumour effect against cultured tumour cells (Kumar *et al*, 2003). Whether or not these compounds exert their antitumour effect solely via inhibition of the p53–MDM2 interaction still needs to be demonstrated (Kumar *et al*, 2003).

Using computer-aided design, Zhao *et al* have synthesised putative nonpeptidic polycyclic MDM2 antagonists. Their initial evaluation has shown a moderate affinity for MDM2 and induction of the p53 pathway in tumour cell lines (Zhao *et al*, 2002). The fungal cyclic nonapeptide Chlorofusin (Figure 1C) was identified by screening a library of microbial extracts. Chlorofusin binds to MDM2 and inhibits the p53–MDM2 interaction. Owing to the high *K*_D_ value of 4.6 *μ*M, the complex chemical structure and a high molecular mass Chlorofusin does not represent a candidate drug but might serve as a lead structure for the design of more potent analogues (Duncan *et al*, 2001, 2003). Several small-molecule antagonist of MDM2 have appeared in recently published patents. However, the limited functional data provided does not allow evaluation of their significance as novel MDM2 antagonists. In summary, the small-molecule p53–MDM2 binding inhibitors described in the literature so far have shown modest potency, lack of selectivity and inadequate pharmacological properties.

Recently, we have identified the first potent and selective small-molecule antagonists of the p53–MDM2 interaction, the Nutlins (Vassilev *et al*, 2004). These *cis*-imidazoline derivatives bind tightly into the p53 pocket of MDM2 and displace p53 from its complexes with MDM2 *in vitro* with IC_50_ in the 100–300 nM range. The crystal structure of MDM2–Nutlin complexes revealed that Nutlins project functional groups into the binding pocket that mimic to a high degree the interaction of the three p53 amino acids critical for the interaction: Phe^19^, Trp^23^ and Leu^26^. Figure 2

**Figure 2:**
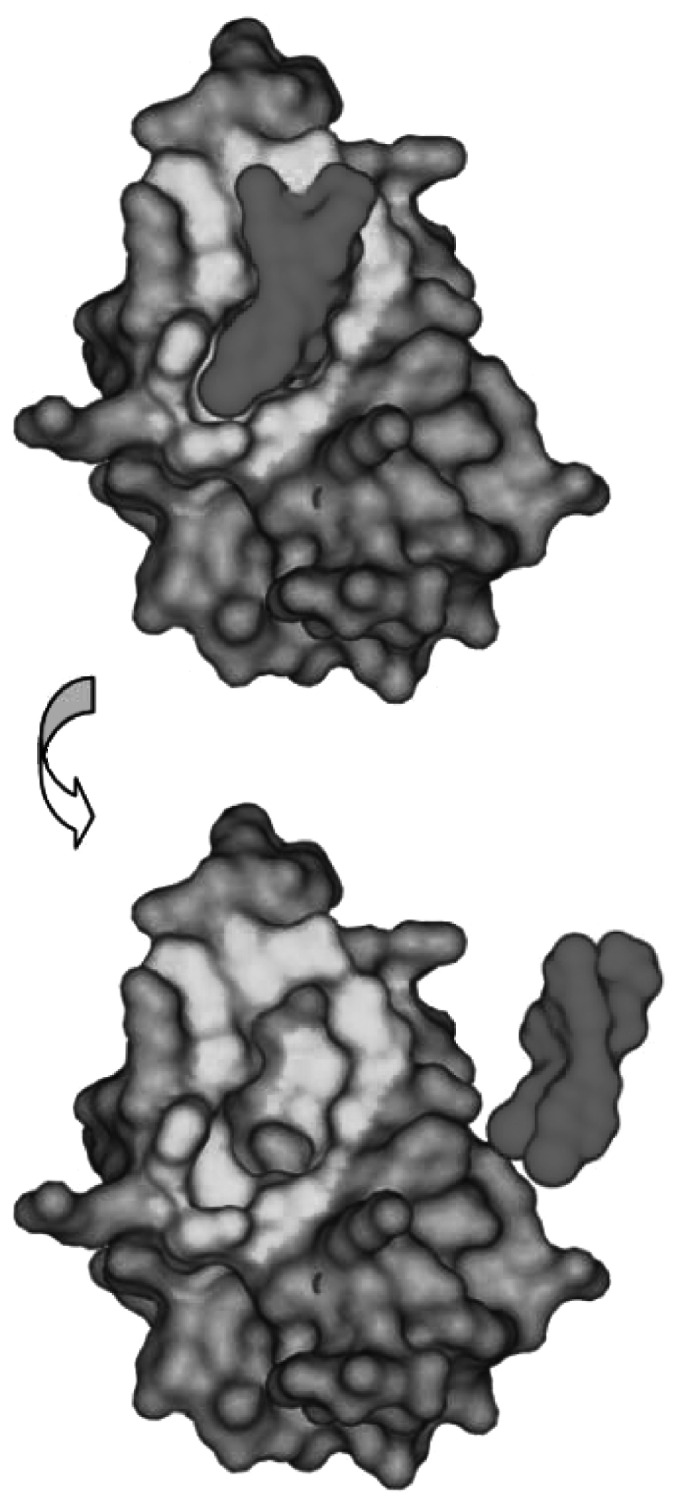
Nutlins bind MDM2 at the p53-binding pocket. Surface representation of MDM2–Nutlin-2 interaction. The p53 binding area within 6 Å distance from Nutlin-2 is depicted in yellow and Nutlin-2 in green. Nutlin-2 is rotated out of the p53-binding pocket in the lower panel. Coordinates are derived from the crystal structure of MDM2–Nutlin-2 complex (Vassilev *et al*, 2004).

shows the high steric complementarity with which the Nutlins bind into the hydrophobic p53-binding pocket on MDM2. Nutlins penetrated cell membranes and inhibited p53–MDM2 binding, leading to stabilisation of p53 and activation of p53 target genes (Stommel and Wahl, 2004; Vassilev *et al*, 2004). The activation of the p53 pathway in cancer cells was manifested by cell cycle arrest in G1 and G2 phase and caspase-dependent apoptosis. Nutlins showed remarkable selectivity for the p53–MDM2 interaction. Their antitumour effect was observed only in cells with wild-type p53 but not in cells with mutant or deleted p53, suggesting that Nutlin activity is derived from activation of the p53 pathway. Interestingly, the occupation of the p53-binding pocket on MDM2 by the Nutlins appears sufficient to disrupt the complex of full-length p53 and MDM2 *in vitro* and *in vivo* independent of the recently suggested additional interaction site between the two proteins (Shimizu *et al*, 2002). Pharmacological properties of the Nutlins allowed the compounds to achieve adequate exposure levels in mouse plasma through the desirable oral route of administration and permitted us to perform proof-of-concept studies using mouse xenograft models of human cancer. Treatment of established human osteosarcoma xenografts (SJSA-1) with nontoxic doses of Nutlin-3 for 3 weeks led to 90% inhibition of tumour growth compared to vehicle controls. Since proliferating mouse fibroblasts appear equally sensitive to MDM2 antagonists as cultured human cells, lack of apparent toxicity during the course of treatment suggests that normal and cancer cells may have different tolerability to elevated level of p53 (Vassilev, 2004). Additional studies with histopathological examination of multiple mouse tissues are needed to assess the potential side effects of this p53-activating approach.

## CONCLUSIONS

The interaction between the master tumour suppressor p53 and its negative regulator MDM2 has been in the focus of cancer drug discovery for nearly a decade. Multiple studies have validated the concept that disrupting this protein–protein interaction can activate the p53 pathway in cancer cells. The identification of the first potent and selective small-molecule MDM2 antagonists, the Nutlins, made it possible to perform *in vivo* validation studies and strengthen the notion that targeting the p53–MDM2 interaction can provide a potentially viable strategy for treating cancer. However, many questions need to be answered before we can understand the true utility of MDM2 antagonists in cancer therapy. Although as many as 50% of all human tumours have retained wild-type p53, and should be thus sensitive to p53-activating therapy, the response rate will most likely be limited by defects in the p53 pathway downstream of p53. Our current state of knowledge points to tumours with wild-type p53 and MDM2 gene amplification as the most likely responders of therapy with MDM2 antagonists. It is believed that in these tumours MDM2 overexpression is the only aberration, thus the restoration of p53 function should lead to an effective apoptotic response. Preclinical studies, now in progress, are aimed at addressing the role of MDM2 status and the genetic background of other members of the p53 pathway in the response to p53 activation by Nutlins.

In summary, the discovery of Nutlins as potent MDM2 antagonists with *in vitro* an *in vivo* activity have provided compelling evidence that the p53–MDM2 interaction represents a tractable target for pharmacological intervention. Given the plethora of data supporting the therapeutic concept of p53 activation, there seems to be a realistic chance that the quest for small-molecule MDM2 antagonists can translate into a clinical benefit in the near future.
